# Health Care Social Robots in the Age of Generative AI: Protocol for a Scoping Review

**DOI:** 10.2196/63017

**Published:** 2025-04-14

**Authors:** Paul Notger Lempe, Camille Guinemer, Daniel Fürstenau, Corinna Dressler, Felix Balzer, Thorsten Schaaf

**Affiliations:** 1 Institute of Medical Informatics Charité – Universitätsmedizin Berlin, corporate member of Freie Universität Berlin & Humboldt–Universität zu Berlin Berlin Germany; 2 School of Business & Economics Freie Universität Berlin Berlin Germany; 3 Medical Library Charité – Universitätsmedizin Berlin, corporate member of Freie Universität Berlin & Humboldt–Universität zu Berlin Berlin Germany

**Keywords:** robotics, social robots, artificial intelligence, generative AI, human-robot interaction, health care sector, PRISMA

## Abstract

**Background:**

Social robots (SR), sensorimotor machines designed to interact with humans, can help to respond to the increasing demands in the health care sector. To ensure the successful use of this technology, acceptance is paramount. Generative artificial intelligence (AI) is an emerging technology with the potential to enhance the functionality of SR and promote user acceptance by further improving human-robot interaction.

**Objective:**

We present a protocol for a scoping review of the literature on the implementation of generative AI in SR in the health care sector. The aim of this scoping review is to map out the intersection of SR and generative AI in the health care sector; to explore if generative AI is applied in SR in the health care sector; to outline which models of generative AI and SR are used for these implementations; and to explore whether user acceptance is reported as an outcome following these implementations. This scoping review supports future research by providing an overview of the state of connectedness of 2 emerging technologies and by mapping out research gaps.

**Methods:**

We follow the methodological framework developed by Arksey and O'Malley and the recommendations by the Joanna Briggs Institute. Our protocol was drafted using the PRISMA-ScR (Preferred Reporting Items for Systematic Reviews and Meta-analyses extension for Scoping Reviews). We will conduct a systematic literature search of the online databases MEDLINE, Embase, CINAHL (Cumulative Index to Nursing and Allied Health Literature), Web of Science, and IEEE Xplore, aiming to retrieve relevant data items via tabular data charting from references meeting specific inclusion criteria which are studies published from 2010 onwards, set in the health care sector, focusing on SR with physical bodies and implemented generative AI. There are no restrictions on study types. Results will be categorized, clustered, and summarized using tables, graphs, visual representations, and narratives.

**Results:**

After conducting a preliminary search and deduplication in the second quarter of 2024, we retrieved 3176 preliminary results. This scoping review will be supplemented with the next methodological steps, including retrieving the results in a reference management tool as well as screening titles, abstracts, and full text regarding specific inclusion criteria. The completion of these steps is scheduled for the second quarter of 2025. Limitations based on the heterogeneity of the included studies and the general breadth of a scoping review compared to a systematic review are to be expected. To reduce bias, we adopted a system of dual reviews and thorough documentation of the study selection.

**Conclusions:**

The conducted preliminary search implies that there are a sufficient number of heterogeneous references to complete this scoping review. To our knowledge, this is the first scoping review on generative AI in health care SR.

**International Registered Report Identifier (IRRID):**

PRR1-10.2196/63017

## Introduction

### Background

Social robots (SR) are interactive robots designed to engage with humans in a social or collaborative manner, often mimicking human behaviors, communication, and social cues [1-3]. SR are already being used in geriatric care, rehabilitation, and the care of people with cognitive disabilities [4]. Since much progress has been made regarding the development of SR in recent years [5], it is to be expected that they will find widespread use in the health care sector in the next few years [6]. Between 2005 and 2010, 1300 units of “Paro,” an artificial seal for therapy of patients with dementia [7], were sold in Japan [8]; as of 2013, “Paro” was adopted in 80% of local care institutes in Denmark [9]. These examples illustrate that SR could be a way of responding to the needs of people in need of care and of filling the gap between the supply and demand of health care professionals. Other examples for SR that can be applied in the health care sector are “Cruzr” [10], a service robot [7,10]; “NAO” [11], a household robot [7,11]; or “Lio” for older adult care [7,12].

For SR to be effectively integrated and successful, their acceptance is paramount. Simply introducing these innovative robots is not sufficient if individuals are hesitant or unwilling to interact with them [13]. Lum [14] emphasizes that the fundamental challenge for the practical application of SR is the willingness of people to accept them in their everyday lives. In democratically constituted, liberal societies, acceptance is deemed a factor for a successful take-up of a technology by the population [15]. While positive effects such as assistance with everyday tasks [16,17], maintaining of individual autonomy [17-19], or increase of happiness [18] are associated with the use of SR in older adult care, their acceptance is noted to vary [6], with acceptance toward the SR depending on the area of their application [15]. With the ongoing development and practical use of SR, the question of their acceptance as a possible component of people’s everyday routines moves further into focus [6,14].

Dialog management is positioned as one of the main goals of human-robot interaction (HRI) [20], increasing user satisfaction and task efficiency [20,21]. In this context, generative artificial intelligence (AI) like OpenAI’s ChatGPT [22], being extensively trained on conversational dialog [23,24], could be used to translate instructions given in natural language into actions executable by a SR [25,26], improving multiparty interactions [21]. Since late 2022, there has been a significant growth in the field of generative AI [27], a technology which will have an impact on all industry sectors with the potential to change how people work and to increase productivity [28]. We believe that by implementing generative AI in SR, usability and user acceptance, key elements of HRI, could be vastly increased. Speech, gesture, mimic recognition, robot customizability, intuitive operation, and interaction among others, are aspects we believe to highly benefit from the potential of generative AI. We expect this emerging technology to largely influence the use and functionality of SR, facilitating their widespread use in the health care sector. Health care professionals, patients, and other persons in need of care could benefit from such advanced robots fulfilling a wide array of valuable tasks. With this scoping review, the authors intend to map out if and how these emerging technologies are interconnected.

### Definitions and Terms

#### Social Robots

Sarrica et al [29] point out that there is a wide array of various definitions for SR, which are partially built on existing definitions or introduce new concepts. For example, Bendel [7] refers to SR as sensorimotor machines explicitly designed to be used in the immediate vicinity of and for direct interaction with humans. A machine is defined as an assemblage of parts that transmit forces, motion, and energy to one another in a predetermined manner [30], consequently implying the necessity of a physical body as well as the presence of sensors and motors for interaction with and perception of the environment by a robot in a narrow sense. While Bendel [7] presents the option to broaden the concept of robots and include software robots like chatbots within SR by relativizing the sensorimotor and physical components, scientific definitions overall tend to describe SR as semiautonomous or autonomous physical bodies, with “machines” and “physical” being some of the main dimensions underlying different definitions [29]. As Kuipers et al [31] point out, the creation of robots with a human-like competence of real-world manipulation is a major challenge of research, since the ability to physically change the surrounding world is of utmost relevance to humans [32]. In the context of care, the burden of physical work is high and further increasing [33,34]. Against this background, we consider only SR with a physical body for this review to ensure a high level of comparability of the results.

#### Generative AI

Generative AI is a type of AI able to generate content [28]. It is based on different kinds of underlying models, for example, generative adversarial networks, encoder-decoder networks, neural networks, or transformers [27]. Subtypes of generative AI are large language models, deep neural networks which are pretrained with millions of parameters on large amounts of unlabeled text [35]. This training enables them to learn the relationship between words or portions of words [28]. Unlike decision tree models, which use algorithms to create predictions or rules based on incoming data, generative AI uses specific algorithms trained on large amounts of data to generate new, previously unseen content [27]. This generated content can be manifold, for example, human-like texts, images, and music [27]. Compared to a conversational AI, which can provide users with human-like responses in a conversation, generative AI can not only generate such responses but generate the content of these responses as well [36]. These responses can even surpass the original programming of the generative AI [36]. Since late 2022, the field of generative AI has grown significantly [27,37]; pretrained and then task-specifically modified large language models became the dominant pattern in the field of natural language processing [38-44], with examples being Google’s BERT (Bidirectional Encoder Representations from Transformers) [38] and OpenAI’s GPT-3 [39]. Generative AI gained increasing public focus since the release of ChatGPT in late 2022, while the concept of generative models of AI began to be recognized since the 2010s [27]. One of the goals in the development of ChatGPT was to facilitate a better reciprocal understanding of the communication between human users and robots [45].

#### Acceptance

Acceptance, acceptability, adoption as well as other related terms in the context of technology acceptance are often used interchangeably [46], describing the same overall concept. Acceptability is described as the user’s willingness toward the use of a system [47]. User acceptance can be defined as “the demonstrable willingness within a user group to employ (information technology) for the tasks it is designed to support“ [48]. The aforementioned terms are occasionally used exchangeable with other concepts of human-computer interaction like user satisfaction [49]. User acceptance can be specified as the users’ intention to use a future technology, their willingness to integrate it into their daily life [50], and the prospective judgment of this new technology [51]. Beetz et al [15], specify acceptance of assistive robotics as being based on trust. Human-related aspects such as cultural background, living conditions, or expectations toward the functionality of a robot as well as criteria of the robot itself such as design [6,32], language capability, or range of functions are relevant for the acceptance of an SR [6]; specifically, an anthropomorphic approach on the design of SR is deemed to be of high relevance [14]. In the literature, the terms facilitators and barriers are commonly used to describe factors of technology uptake in general, for example, facilitators of the implementation of SR can be mobility aspects [52], being physically accessible [52,53], or offering an economic advantage [54]. Davis [55] developed the Technology Acceptance Model to describe why a person does or does not use a technology. A person’s attitude toward using a technology depends on the technology’s perceived usefulness and its perceived ease of use, the behavioral intention to use a technology depends on the aforementioned attitude toward using and the perceived usefulness directly [55]. Thus, this model implicates a complex relationship between the user’s subjective ability to accept, and the quality of a technology of being acceptable. Based on a revision of this model [56], Heerink et al [57], proposed the Almere Model for technology acceptance of SR by older adult users, examining 12 areas related to HRI: anxiety, attitude toward technology, facilitating conditions, intention to use, perceived adaptiveness, perceived enjoyment, perceived ease of use, perceived sociability, perceived usefulness, social influence, social presence, and trust [57]. The Almere Model integrates the multi-dimensionality of acceptance formation by relating robot-specific factors to their subjective perception by humans.

### Literature Gap

To address the intersection of SR and generative AI, we conducted a preliminary search for systematic reviews, scoping reviews, or protocols for reviews in the online databases MEDLINE, Embase, CINAHL, Web of Science, and IEEE Xplore based on our search strategy presented in stage 2. For example, some of these reviews focus on AI in the health care sector in general [58-101]; other reviews focus on social or surgical robots in the health care sector in general [102-118]; a few highlight the use of AI in surgical robots [119,120] or the use of chatbots [80,88,121] in the health care sector; Clabaugh and Matarić [122] conducted a systematic review on enablers of autonomy of SR; Huang et al [123], conducted a systematic review on antecedents and consequences of the application of intelligent SR; Hung et al [124], published a protocol for a systematic review on the facilitators and barriers to the use of robots equipped with AI in older adult care; Lee et al [125] conducted a systematic review and meta-analysis on the effect of AI-equipped SR on cognitive function.

However, none of these reviews focus specifically on the implementation of generative AI in SR in the health care sector and the interconnectedness of these 2 emerging technologies.

### Aim

The aim of this scoping review is to map out if and how 2 emerging technologies: generative AI and SR are interconnected. This scoping review is based on an overview of relevant publications that have considered the implementation of generative AI in physical health care SR. We intend to explore whether generative AI is applied in SR in the health care sector, what models of generative AI and SR are used for this implementation, in which setting these implementations take place, and if user acceptance is among the reported outcomes following these implementations.

## Methods

### Study Design

In this review, we follow the methodological framework developed by Arksey and O’Malley [126] and the recommendations by the Joanna Briggs Institute [127]. We apply the following stages here: (1) identifying the research question, (2) identifying relevant studies, (3) selection of studies, (4) data charting, and (5) collating, summarizing, and reporting the results. To report our results, we use the PRISMA-ScR (Preferred Reporting Items for Systematic Reviews and Meta-Analyses extension for Scoping Reviews), attached in (Multimedia Appendix 1), and, where applicable, PRISMA-P (Preferred Rpeorting Items for Systematic Reviews and Meta-Analyses Protocols; Multimedia Appendix 2).

### Research Team

The research team conducting this review consists of a medical student researcher interested in the fields of medical sociology and medical data science, PL; a researcher with a background in health economics, CG; a professor for digitalization working in the field of digital transformation and applications, DF; the head of information literacy education and systematic review, medical library, CD; a professor for medical data science and an expert on human-computer interaction, FB; and a senior researcher working in the field of medical informatics and health care organization and innovation, TS.

### Stage 1: Identifying the Research Question

The main research questions of this protocol are as follows: (1) Are generative AI models implemented in SR in the health care sector? The following subquestions are posed: (2) Which models of generative AI and SR are used for these implementations? (3) What are the settings of these implementations? (4) Has the acceptance of SR been reported as an outcome following these implementations?

Answering these questions can support future research by mapping out potential research gaps and providing an overview of the state of connectedness of 2 emerging technologies, SR, and generative AI.

### Stage 2: Identifying Relevant Studies

To identify relevant studies, a systematic literature search of the electronic databases MEDLINE (via Ovid; 2010 onwards), Embase (via Ovid; 2010 onwards), CINAHL (via EBSCOhost; 2010 onwards), Web of Science (2010 onwards), and IEEE Xplore (2010 onwards) will be conducted. The selection of these databases follows the recommendation of a member of the research team (CD), an information specialist of the medical library of Charité, covering a broad and interdisciplinary field including computer science, social and behavioral sciences, and medicine. To construct our search queries, we followed the guidelines of the Peer Review of Electronic Search Strategies (PRESS) [128]. Textbox 1 presents the main search terms underlying these search queries. An initial search query was built with Ovid for MEDLINE. The online tool Polyglot Search Translator [129] is used to adapt this search query for the databases Embase, CINAHL, and Web of Science. For IEEE Xplore, the initial search query is adapted manually to account for the different syntax and limit of search terms per search clause. Duplicates will be deleted by using the online tool Systematic Review Accelerator [130] after the corresponding results have been documented and managed using the reference managing software EndNote (Clarivate).

The search terms are based on the expertise of the research team, an internal focus group, and an additional use of the AI chatbot, ChatGPT (OpenAI) to detect further relevant synonyms and terms. These terms are combined using the appropriate Boolean operators AND Specialty OR to generate the search queries. The exact search queries for MEDLINE, Embase, CINAHL, Web of Science, and IEEE Xplore are referenced in (Multimedia Appendix 3).

**Overview of the main search terms.**

**Social robot**

- Social robot
- Interactive robot
- Companion robot
- Humanoid robot
- Personal robot
- Emotional robot
- Assistive robot
- Service robot
- Communicative robot
- Socially assistive robot
- Socially interactive robot
- Care robot
- Household robot
- Pleasure robot
- Sex robot
- Anthropomorphic robot
- Autonomous robot
- Therapy robot
- Entertainment robot
- Robotics

**Generative AI**

- Generative AI
- Generative artificial intelligence
- Artificial intelligence
- AI
- Large Language Model
- LLM
- Foundation Model
- Deep Neural Network
- Machine Learning
- ChatGPT
- OpenAI
- YouChat
- Stable Diffusion
- DALL-E
- Runway
- Midjourney
- MusicLM
- VALL-E
- ElevenLabs
- Codex
- AlphaCode
- GitHub Copilot
- Emotion recognition
- Speech recognition
- Gesture recognition
- Facial expression analysis

**Health care sector**

- Health care
- Health care Sector
- Health service
- HCS
- Medical Sector
- Hospital
- Clinic
- Nursing home
- Rehabilitation
- Primary care
- Speciality care
- Mental health
- Public health
- Long-term care facility
- Physician
- Nursing
- Allied health professions
- Medical practitioner
- Care
- Medical device
- Health informatics
- Patient
- Person in need of care
- Invalid
- Convalescent
- Sick person
- Elderly
- Elderly person
- Elderly care
- Nursing home
- Nursing personnel
- Aged

### Stage 3: Study Selection

The PCC (Population, Concept, and Context) Framework was used as a basis for the inclusion criteria. The population is SR with physical bodies; the concept is the implementation of generative AI in SR; and the context is the health care sector or SR intended for the health care sector. The framework underlying this scoping review is presented in Textbox 2, inclusion and exclusion criteria to be used in this scoping review are highlighted in Textboxes 3 and 4.

The screening process is divided into 2 subsequent steps: an initial title and abstract screening, and a secondary full-text screening. At least 2 reviewers (PL and TS) will conduct the primary title and abstract screening independently, using the eligibility criteria. Then, the secondary full-text screening of all references included in the title and abstract screening will be conducted by at least 2 members of the research team (PL and TS) independently, using the same eligibility criteria. In the secondary screening, disagreements will be resolved by discussion and consensus of 3 members of the research team (PL, TS, and CG). Any restrictions regarding study design, time frame, or language are not intended. A PRISMA (Preferred Reporting Items for Systematic Reviews and Meta-Analyses) flow diagram will be used to show a graphic representation of the process of study selection.

**Overview of the Population, Concept, and Context framework of this review.**

- Population: social robots (SR) with physical bodies.
- Concept: implementation of generative artificial intelligence in SR.
- Context: in the health care sector or SR intended for the health care sector.

**Overview of inclusion criteria.**

- Studies focusing on social robots (SR) with physical bodies are considered.
- Generative artificial intelligence must be implemented in these SR.
- Studies set in the health care sector are considered, or studies focusing on SR intended to be used in the health care sector.
- Studies published since 2010 are considered, as the concept of generative models of artificial intelligence began to be recognized in the 2010s [27].
- No restrictions concerning study design (ie, reviews or case studies are included), time frame, or language

**Overview of exclusion criteria.**

- Social robots without physical bodies are excluded (ie, chatbots or artificial intelligence avatars).
- Surgical robots are excluded.
- Studies published before 2010 are not considered.
- Studies focusing on social robots outside of or not intended for the health care sector are not considered.

### Stage 4: Data Charting

The variables that are to be extracted are based on recommendations by 2 members of the research team (DF and FB). We extract data describing the specifications of the applied SR and model of the implemented generative AI, the characteristics of this Implementation and HRI, as well as general information regarding for example, but not limited to, studies’ populations, reported outcomes, or country of origin. Textbox 5 conceptualizes the items we aim to extract from the included studies.

The authors will create preliminary data charting forms in Microsoft Excel, which will undergo independent pilot testing using a sample of publications (for example 10 articles). After achieving consistent results and obtaining approval from at least 2 researchers of the team (DF, FB), data charting will be conducted by one team member (PL) for all included full-text articles. A total of 2 other team members (CG and TS) will verify the charted data to ensure the inclusion of all relevant information. A draft of a data charting form is attached in (Multimedia Appendix 4). A team member (CD) will provide support for this process.

**Overview of data extraction items.**

**Items and description**

- Author and year: name of first author, year of publication.
- Study type: What type of study was conducted (eg, randomized controlled trial, cohort study, case-control study, qualitative study, etc)?
- Study design: framework, methods, and procedures applied to answer the research question.
- Origin: where the study was conducted.
- Population and sample size: which and how many participants were in the study.
- Aim: purpose of the study.
- Make of social robot (SR): model and developer of SR.
- Shape of SR: traits of the physical body of SR (eg, number of appendices, anthropomorphic or animal-like shape).
- Mobility of SR: ability of SR to move.
- Autonomy of SR: whether the SR is autonomous or controlled remotely.
- Interface of SR: means of communication or interaction with SR (eg, speech, display, touchscreen, gestures, facial expressions, emotion recognition, remote control, haptic, etc).
- Human-robot interaction: specific traits of interaction with SR (eg, duration, tasks, and intention).
- Model of generative artificial intelligence (AI): specific model of implemented generative AI.
- Use of generative AI: what the implemented generative AI is used for.
- Element of health care sector: sub-sector of the health care sector in which SR was applied (eg, inpatient or outpatient sector, nursing home, day care, home care, etc).
- Outcome acceptance: if user acceptance has been reported as a study outcome.
- Acceptance measurement: tools or models used to measure user acceptance.

### Stage 5: Collating, Summarizing, and Reporting the Results

The studies will be collated, categorized, summarized, and reported based on the data items extracted in the former step, for example, the type of SR, the context of HRI, the use of generative AI, the country of origin, derived acceptance-promoting aspects, etc. To synthesize the results, we will cluster similar publications by classifying the collected data items. The findings of this synthesis will be presented through a series of adequate tables, graphs, and visual representations, as well as corresponding narrative summaries. While we intend to use the main categories of data items from the former steps, we anticipate and remain open to discovering further categories, as well as expanding or modifying existing ones, as this review progresses. A team member (CD) will provide guidance for this stage.

## Results

**Figure 1:**
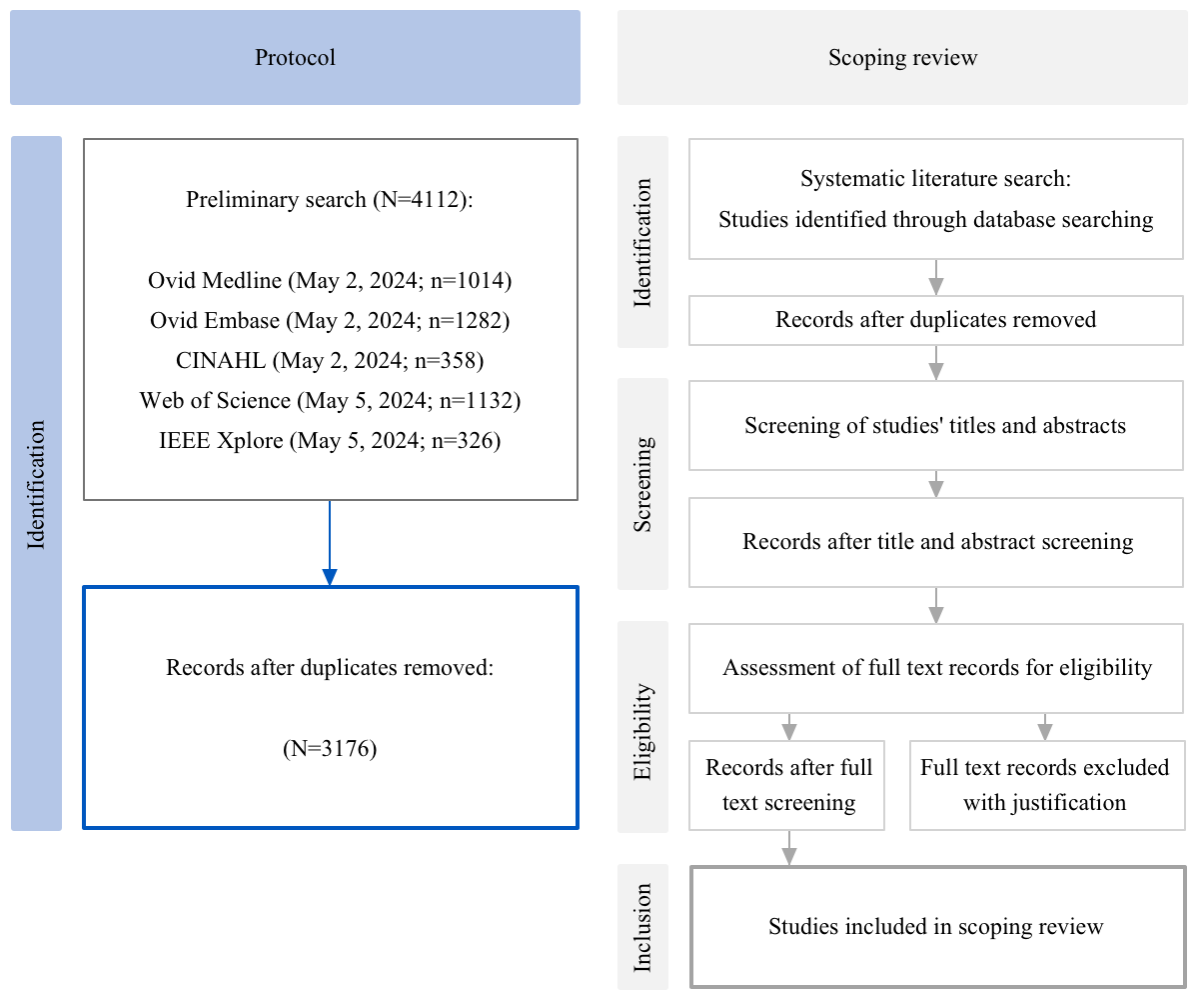
Flow diagram of this protocol’s preliminary search and subsequent scoping review.

A preliminary literature search was conducted in the second quarter of 2024 to screen relevant existing literature as well as to verify no other scoping reviews of the same focus have been published. This preliminary search of the electronic databases MEDLINE, Embase, CINAHL, Web of Science, and IEEE Xplore was based on the search strategy presented in Stage 2 of this protocol. In this preliminary search, we retrieved a total of 4112 references, of which 3176 remained after deduplication. Figure 1 presents a flow diagram of our preliminary search for this protocol, and of the subsequent scoping review. Following these preliminary results, we deem the literature sufficient to conduct this scoping review. To our knowledge, this is the first scoping review on the implementation of generative AI in Health care SR. We intend to complete the following methodological steps of the scoping review by the second quarter of 2025.

## Discussion

### Principal Findings

The authors anticipated to retrieve relevant studies, gaining an overview of scientific literature to decide if it is feasible to conduct a scoping review on this topic. We anticipated the results to be sufficient in number, since generative AI and SR are emerging technologies of high relevance. The preliminary search provided 3176 results after deduplication. Based on these preliminary results, we anticipate that there is sufficient literature to conduct this scoping review. To our knowledge, no other reviews of the same scope have been published.

### Limitations

Based on the methodology of this protocol, some limitations can be derived. The authors aim to synthesize results from publications that are expected to have widely heterogeneous populations, origins, outcomes, and overall methodologies. For example, different age groups or cultural backgrounds of participants limit our ability to generalize the findings of this review. In general, scoping reviews tend to be inherently limited by focusing on breadth rather than depth [131]. Also, the selection of databases, search terms, inclusion criteria, and studies based on the research team’s experience might pose a source of possible bias. To reduce overall bias, we adopted a system of dual reviews, discussing conflicting results with senior researchers of the team, and thoroughly documenting the process of study selection.

### Conclusions

This scoping review on the interconnectedness of 2 emerging technologies will outline the implementation of generative AI in health care SR, and to map out and categorize which models of generative AI and SR are used in these implementations. Based on relevant literature and the expertise of the research team, it is the authors’ intention to answer the primary and secondary research questions: (1) Are generative AI models implemented in SR in the health care sector? (2) Which models of generative AI and SR are used for these implementations? (3) What are the settings of these implementations? (4) Has the acceptance of SR been reported as an outcome following these implementations? While there are previous reviews on either AI or SR in general in the health care sector, there is merely a scant number of reviews on AI-equipped social or surgical robots, and there are, to our knowledge, no other reviews of the same scope as our review. Conducting this scoping review supports future research by mapping out further research gaps and by providing an overview of the state of the connectedness of SR and generative AI, a relatively new technology with vast potential.

## Appendix

Multimedia Appendix 1 PRISMA-ScR checklist.

Multimedia Appendix 2 PRISMA-P checklist.

Multimedia Appendix 3 Search queries for MEDLINE, Embase, CINAHL, Web of Science, and IEEE Xplore.

Multimedia Appendix 4 Draft of a data charting form.

## Abbreviations

AI: artificial intelligence
BERT: Bidirectional Encoder Representations from Transformers
HRI: human-robot interaction
PRESS: Peer Review of Electronic Search Strategies
PRISMA: Preferred Reporting Items for Systematic Reviews and Meta-Analyses
PRISMA-P: Preferred Reporting Items for Systematic Review and Meta-Analysis Protocols
PRISMA-ScR: Preferred Reporting Items for Systematic Reviews and Meta-Analyses extension for Scoping Reviews
SR: social robot

## Data Availability

The datasets generated or analyzed during this study are available from the corresponding author on reasonable request.
