# Supplementary material for: Health Care Social Robots in the Age of Generative AI: Protocol for a Scoping Review
Source: JMIR Res Protoc. 2025 Apr 14;14:e63017. doi: 10.2196/63017 (PMC12038295; doi:10.2196/63017)
Supplement: Multimedia Appendix 3 [file resprot_v14i1e63017_app3.docx]

### Ovid Medline:

# social robots

1 (socia* adj robot*).ab,ti.

2 (interactive adj robot*).ab,ti.

3 (companion adj robot*).ab,ti.

4 (humanoid adj robot*).ab,ti.

5 (personal adj robot*).ab,ti.

6 (emotional adj robot*).ab,ti.

7 (assistive adj robot*).ab,ti.

8 (service adj robot*).ab,ti.

9 (communicative adj robot*).ab,ti.

10 (social* adj assistive adj robot*).ab,ti.

11 (social* adj interactive adj robot*).ab,ti.

12 (care adj robot*).ab,ti.

13 (household adj robot*).ab,ti.

14 (pleasure adj robot).ab,ti.

15 (sex adj robot*).ab,ti.

16 (anthropomorphic adj robot*).ab,ti.

17 (autonomous adj robot*).ab,ti.

18 (therap* adj robot*).ab,ti.

19 (entertainment adj robot*).ab,ti.

20 Robotics/

21 Robotics.mp.

22 Robotic Surgical Procedures/ or Robotic Surgical Procedures.mp.

# generative ai

23 (Generative adj AI).ab,ti.

24 (Generative adj artificial adj intelligence$1).ab,ti.

25 (artificial adj intelligence$1).ab,ti.

26 "AI".ab,ti.

27 (Large adj Language adj Model$1).ab,ti.

28 "LLM*".ab,ti.

29 (Foundation adj Model$1).ab,ti.

30 (Deep adj Neural adj Network$1).ab,ti.

31 (Machine adj Learning).ab,ti.

32 ChatGPT.ab,ti.

33 GPT.ab,ti.

34 OpenAI.ab,ti.

35 YouChat.ab,ti.

36 (Stable adj Diffusion).ab,ti.

37 DALL-E.ab,ti.

38 Runway.ab,ti.

39 Midjourney.ab,ti.

40 MusicLM.ab,ti.

41 VALL-E.ab,ti.

42 ElevenLabs.ab,ti.

43 Codex.ab,ti.

44 AlphaCode.ab,ti.

45 (GitHub adj Copilot).ab,ti.

46 (emotion adj recogni*).ab,ti.

47 (speech adj recogni*).ab,ti.

48 (gesture adj recogni*).ab,ti.

49 (facial adj expression adj analys*).ab,ti.

50 Artificial Intelligence/

51 Artificial Intelligence.mp.

52 Neural Networks, Computer/

53 Neural Networks, Computer.mp.

54 Deep Learning/

55 Deep Learning.mp.

56 Machine Learning/

57 Machine Learning.mp.

# healthcare sector

58 Healthcare.ab,ti.

59 (Healthcare adj Sector).ab,ti.

60 (Health adj Care).ab,ti.

61 (Health adj Care adj Sector).ab,ti.

62 (Health adj Service$1).ab,ti.

63 HCS.ab,ti.

64 (Medical adj Sector).ab,ti.

65 Hospital$1.ab,ti.

66 Clinic*.ab,ti.

67 (Nursing adj home$1).ab,ti.

68 "rehabilitation*".ab,ti.

69 (primary adj care).ab,ti.

70 (speciality adj care).ab,ti.

71 (mental adj health*).ab,ti.

72 (public adj health*).ab,ti.

73 (long-term adj care adj facilit*).ab,ti.

74 physician$1.ab,ti.

75 "nurs*".ab,ti.

76 (allied adj health adj profession*).ab,ti.

77 (medical adj practitioner*).ab,ti.

78 "care*".ab,ti.

79 (medical adj device$1).ab,ti.

80 (health adj informatics).ab,ti.

81 "patient$1".ab,ti.

82 (person$1 adj in adj need adj of adj care).ab,ti.

83 invalid$1.ab,ti.

84 "convalescent$1".ab,ti.

85 (sick adj person$1).ab,ti.

86 "elder*".ab,ti.

87 (elderly adj person$1).ab,ti.

88 (elderly adj care).ab,ti.

89 (nursing adj home$1).ab,ti.

90 (nursing adj personnel).ab,ti.

91 Health Care Sector/

92 Health Care Sector.mp.

93 Health Personnel/

94 Health Personnel.mp.

95 Patients/

96 Patients.mp.

97 Aged/

98 Aged.mp.

99 "Health Services for the Aged"/

100 "Health Services for the Aged".mp.

101 Nursing Homes/

102 Nursing Homes.mp.

103 Caregivers/

104 Caregivers.mp.

105 1 or 2 or 3 or 4 or 5 or 6 or 7 or 8 or 9 or 10 or 11 or 12 or 13 or 14 or 15 or 16 or 17 or 18 or 19 or 20 or 21

106 23 or 24 or 25 or 26 or 27 or 28 or 29 or 30 or 31 or 32 or 33 or 34 or 35 or 36 or 37 or 38 or 39 or 40 or 41 or 42 or 43 or 44 or 45 or 46 or 47 or 48 or 49 or 50 or 51 or 52 or 53 or 54 or 55 or 56 or 57

107 58 or 59 or 60 or 61 or 62 or 63 or 64 or 65 or 66 or 67 or 68 or 69 or 70 or 71 or 72 or 73 or 74 or 75 or 76 or 77 or 78 or 79 or 80 or 81 or 82 or 83 or 84 or 85 or 86 or 87 or 88 or 89 or 90 or 91 or 92 or 93 or 94 or 95 or 96 or 97 or 98 or 99 or 100 or 101 or 102 or 103 or 104

108 105 and 106 and 107

109 limit 108 to yr="2010 -Current"

### Ovid Embase:

Embase Classic+Embase <1947 to 2024 May 03>

1 (((socia* adj robot*) or (interactive adj robot*) or (companion adj robot*) or (humanoid adj robot*) or (personal adj robot*) or (emotional adj robot*) or (assistive adj robot*) or (service adj robot*) or (communicative adj robot*) or (social* adj assistive adj robot*) or (social* adj interactive adj robot*) or (care adj robot*) or (household adj robot*) or (pleasure adj robot) or (sex adj robot*) or (anthropomorphic adj robot*) or (autonomous adj robot*) or (therap* adj robot*) or (entertainment adj robot*)).tw. or Robotics/ or Robotics.mp.) and (((Generative adj AI) or (Generative adj artificial adj intelligence$1) or (artificial adj intelligence$1) or AI or (Large adj Language adj Model$1) or LLM* or (Foundation adj Model$1) or (Deep adj Neural adj Network$1) or (Machine adj Learning) or ChatGPT or GPT or OpenAI or YouChat or (Stable adj Diffusion) or DALL-E or Runway or Midjourney or MusicLM or VALL-E or ElevenLabs or Codex or AlphaCode or (GitHub adj Copilot) or (emotion adj recogni*) or (speech adj recogni*) or (gesture adj recogni*) or (facial adj expression adj analys*)).tw. or "Artificial Intelligence"/ or "Artificial Intelligence".mp. or "Neural Networks, Computer"/ or "Neural Networks, Computer".mp. or "Deep Learning"/ or "Deep Learning".mp. or "Machine Learning"/ or "Machine Learning".mp.) and ((Healthcare or (Healthcare adj Sector) or (Health adj Care) or (Health adj Care adj Sector) or (Health adj Service$1) or HCS or (Medical adj Sector) or Hospital$1 or Clinic* or (Nursing adj home$1) or rehabilitation* or (primary adj care) or (speciality adj care) or (mental adj health*) or (public adj health*) or (long-term adj care adj facilit*) or physician$1 or nurs* or (allied adj health adj profession*) or (medical adj practitioner*) or care* or (medical adj device$1) or (health adj informatics) or patient$1 or (person$1 adj in adj need adj of adj care) or invalid$1 or convalescent$1 or (sick adj person$1) or elder* or (elderly adj person$1) or (elderly adj care) or (nursing adj home$1) or (nursing adj personnel)).tw. or "Health Care Sector"/ or "Health Care Sector".mp. or "Health Personnel"/ or "Health Personnel".mp. or Patients/ or Patients.mp. or Aged/ or Aged.mp. or "Health Services for the Aged"/ or "Health Services for the Aged".mp. or "Nursing Homes"/ or "Nursing Homes".mp. or Caregivers/ or Caregivers.mp.) 1380

2 limit 1 to yr="2010 -Current" 1282

### Web of Science:

# Web of Science Search Strategy (v0.1)

# Database: Web of Science Core Collection

# Entitlements:

- WOS.SCI: 1900 to 2023

- WOS.AHCI: 1975 to 2023

- WOS.ESCI: 2018 to 2023

- WOS.SSCI: 1956 to 2023

# Searches:

1: ((((((((((((((((((((((((((((AB=(patient)) OR AB=(patients)) OR AB=(outpatient)) OR AB=(outpatients)) OR AB=(invalid)) OR AB=(invalids)) OR AB=(convalescent)) OR AB=(convalescents)) OR AB=(sick person)) OR AB=(sick persons)) OR AB=(elder)) OR AB=(elders)) OR AB=(elderly)) OR AB=(nurse)) OR AB=(nurses)) OR AB=(nursing staff)) OR AB=(care-giver)) OR AB=(care-givers)) OR AB=(caregiver)) OR AB=(caregivers)) OR AB=(medical staff)) OR AB=(caretaker)) OR AB=(caretakers)) OR AB=(attendant)) OR AB=(attendants)) OR AB=(therapist)) OR AB=(therapists)) OR AB=(physician)) OR AB=(physicians) Date Run: Thu Apr 06 2023 13:50:50 GMT+0200 (Mitteleuropäische Sommerzeit) Results: 7067889

2: (((((((((((((((((((((((((((((((((((((((AB=(interact)) OR AB=(interaction)) OR AB=(interactions)) OR AB=(communication)) OR AB=(relationship)) OR AB=(human-robot interaction)) OR AB=(human-robot interactions)) OR AB=(human robot interaction)) OR AB=(human robot interactions)) OR AB=(human-computer interaction)) OR AB=(human-computer interactions)) OR AB=(HRI)) OR AB=(HCI)) OR AB=(co-evolution)) OR AB=(user experience)) OR AB=(user experiences)) OR AB=(UX)) OR AB=(user interface)) OR AB=(user interfaces)) OR AB=(user interaction)) OR AB=(user interactions)) OR AB=(user satisfaction)) OR AB=(user-centered design)) OR AB=(user engagement)) OR AB=(customer experience)) OR AB=(customer experiences)) OR AB=(CX)) OR AB=(usability)) OR AB=(user enjoyment)) OR AB=(accept)) OR AB=(acceptance)) OR AB=(adopt)) OR AB=(adoption)) OR AB=(technology acceptance)) OR AB=(technology adoption)) OR AB=(technology implementation)) OR AB=(technology assimilation)) OR AB=(technology incorporation)) OR AB=(technology uptake)) OR AB=(technology embracement) Date Run: Thu Apr 06 2023 16:44:20 GMT+0200 (Mitteleuropäische Sommerzeit) Results: 5282280

3: (((((((((((((((((((((((((((((((AB=(social robot)) OR AB=(social robots)) OR AB=(sociable robot)) OR AB=(sociable robots)) OR AB=(interactive robot)) OR AB=(interactive robots)) OR AB=(companion robot)) OR AB=(companion robots)) OR AB=(humanoid robot)) OR AB=(humanoid robots)) OR AB=(personal robot)) OR AB=(personal robots)) OR AB=(emotional robot)) OR AB=(emotional robots)) OR AB=(assistive robot)) OR AB=(assistive robots)) OR AB=(service robot)) OR AB=(service robots)) OR AB=(communicative robot)) OR AB=(communicative robots)) OR AB=(social assistive robot)) OR AB=(social assistive robots)) OR AB=(socially assistive robot)) OR AB=(socially assistive robots)) OR AB=(care robot)) OR AB=(care robots)) OR AB=(household robot)) OR AB=(household robots)) OR AB=(pleasure robot)) OR AB=(pleasure robots)) OR AB=(anthropomorphic robot)) OR AB=(anthropomorphic robots) Date Run: Thu Apr 06 2023 16:49:43 GMT+0200 (Mitteleuropäische Sommerzeit) Results: 14577

4: #1 AND #2 AND #3 Date Run: Thu Apr 06 2023 16:50:00 GMT+0200 (Mitteleuropäische Sommerzeit) Results: 1384

### IEEE Xplore:

("All Metadata":"social robot" OR "All Metadata":"social robotics" OR "All Metadata":"interactive robot" OR "All Metadata":"companion robot" OR "All Metadata":"humanoid robot" OR "All Metadata":"personal robot" OR "All Metadata":"emotional robot" OR "All Metadata":"assistive robot" OR "All Metadata":"service robot" OR "All Metadata":"communicative robot" OR "All Metadata":"socially assistive robot" OR "All Metadata":"socially interactive robot" OR "All Metadata":"care robot" OR "All Metadata":"household robot" OR "All Metadata":"pleasure robot" OR "All Metadata":"sex robot" OR "All Metadata":"anthropomorphic robot" OR "All Metadata":"autonomous robot" OR "All Metadata":"entertainment robot")

AND

("All Metadata":"generative ai" OR "All Metadata":"generative artificial intelligence" OR "All Metadata":"artificial intelligence" OR "All Metadata":"large language model" OR "All Metadata":"llm" OR "All Metadata":"foundation model" OR "All Metadata":"deep neural network" OR "All Metadata":"machine learning" OR "All Metadata":"gpt" OR "All Metadata":"openai" OR "All Metadata":"youchat" OR "All Metadata":"stable diffusion" OR "All Metadata":"dall-e" OR "All Metadata":"runway" OR "All Metadata":"midjourney" OR "All Metadata":"musiclm" OR "All Metadata":"vall-e" OR "All Metadata":"elevenlabs" OR "All Metadata":"alphacode" OR "All Metadata":"github copilot" OR "All Metadata":"emotion recognition" OR "All Metadata":"speech recognition" OR "All Metadata":"gesture recognition" OR "All Metadata":"facial expression analysis")

AND

("All Metadata":"healthcare" OR "All Metadata":"healthcare sector" OR "All Metadata":"health service" OR "All Metadata":"HCS" OR "All Metadata":"medical sector" OR "All Metadata":"hospital" OR "All Metadata":"clinic" OR "All Metadata":"nursing come" OR "All Metadata":"rehabilitation" OR "All Metadata":"primary care" OR "All Metadata":"speciality care" OR "All Metadata":"mental health" OR "All Metadata":"public health" OR "All Metadata":"long-term care facilit*" OR "All Metadata":"physician" OR "All Metadata":"nurs*" OR "All Metadata":"allied health profession" OR "All Metadata":"medical practitioner" OR "All Metadata":"health informatics" OR "All Metadata":"patient" OR "All Metadata":"person* in need of care" OR "All Metadata":"invalid" OR "All Metadata":"convalescent" OR "All Metadata":"sick person" OR "All Metadata":"elder*")

### Ebsco CINAHL:

(((TI socia* OR AB socia*) W1 (TI robot* OR AB robot*)) ) OR (((TI interactive OR AB interactive) W1 (TI robot* OR AB robot*)) ) OR (((TI companion OR AB companion) W1 (TI robot* OR AB robot*)) ) OR (((TI humanoid OR AB humanoid) W1 (TI robot* OR AB robot*)) ) OR (((TI personal OR AB personal) W1 (TI robot* OR AB robot*)) ) OR (((TI emotional OR AB emotional) W1 (TI robot* OR AB robot*)) ) OR (((TI assistive OR AB assistive) W1 (TI robot* OR AB robot*)) ) OR (((TI service OR AB service) W1 (TI robot* OR AB robot*)) ) OR (((TI communicative OR AB communicative) W1 (TI robot* OR AB robot*)) ) OR (((TI social* OR AB social*) W1 (TI assistive OR AB assistive) W1 (TI robot* OR AB robot*)) ) OR (((TI social* OR AB social*) W1 (TI interactive OR AB interactive) W1 (TI robot* OR AB robot*)) ) OR (((TI care OR AB care) W1 (TI robot* OR AB robot*)) ) OR (((TI household OR AB household) W1 (TI robot* OR AB robot*)) ) OR (((TI pleasure OR AB pleasure) W1 (TI robot OR AB robot)) ) OR (((TI sex OR AB sex) W1 (TI robot* OR AB robot*))) OR (((TI anthropomorphic OR AB anthropomorphic) W1 (TI robot* OR AB robot*)) ) OR (((TI autonomous OR AB autonomous) W1 (TI robot* OR AB robot*)) ) OR (((TI therap* OR AB therap*) W1 (TI robot* OR AB robot*)) ) OR (((TI entertainment OR AB entertainment) W1 (TI robot* OR AB robot*)) ) OR ((MH Robotics)) OR (Robotics)

AND

(((TI Generative OR AB Generative) W1 (TI AI OR AB AI)) ) OR (((TI Generative OR AB Generative) W1 (TI artificial OR AB artificial) W1 (TI intelligence?1 OR AB intelligence?1)) ) OR (((TI artificial OR AB artificial) W1 (TI intelligence?1 OR AB intelligence?1)) ) OR ((TI AI OR AB AI)) OR (((TI Large OR AB Large) W1 (TI Language OR AB Language) W1 (TI Model?1 OR AB Model?1)) ) OR ((TI LLM* OR AB LLM*)) OR (((TI Foundation OR AB Foundation) W1 (TI Model?1 OR AB Model?1)) ) OR (((TI Deep OR AB Deep) W1 (TI Neural OR AB Neural) W1 (TI Network?1 OR AB Network?1)) ) OR (((TI Machine OR AB Machine) W1 (TI Learning OR AB Learning)) ) OR ((TI ChatGPT OR AB ChatGPT)) OR ((TI GPT OR AB GPT)) OR ((TI OpenAI OR AB OpenAI)) OR ((TI YouChat OR AB YouChat)) OR (((TI Stable OR AB Stable) W1 (TI Diffusion OR AB Diffusion)) ) OR ((TI DALL-E OR AB DALL-E)) OR ((TI Runway OR AB Runway)) OR ((TI Midjourney OR AB Midjourney)) OR ((TI MusicLM OR AB MusicLM)) OR ((TI VALL-E OR AB VALL-E)) OR ((TI ElevenLabs OR AB ElevenLabs)) OR ((TI Codex OR AB Codex)) OR ((TI AlphaCode OR AB AlphaCode)) OR (((TI GitHub OR AB GitHub) W1 (TI Copilot OR AB Copilot)) ) OR (((TI emotion OR AB emotion) W1 (TI recogni* OR AB recogni*)) ) OR (((TI speech OR AB speech) W1 (TI recogni* OR AB recogni*)) ) OR (((TI gesture OR AB gesture) W1 (TI recogni* OR AB recogni*)) ) OR (((TI facial OR AB facial) W1 (TI expression OR AB expression) W1 (TI analys* OR AB analys*)) ) OR ((MH "Artificial Intelligence")) OR ("Artificial Intelligence") OR ((MH "Neural Networks, Computer")) OR ("Neural Networks, Computer") OR ((MH "Deep Learning")) OR ("Deep Learning") OR ((MH "Machine Learning")) OR ("Machine Learning")

AND

((TI Healthcare OR AB Healthcare)) OR (((TI Healthcare OR AB Healthcare) W1 (TI Sector OR AB Sector)) ) OR (((TI Health OR AB Health) W1 (TI Care OR AB Care)) ) OR (((TI Health OR AB Health) W1 (TI Care OR AB Care) W1 (TI Sector OR AB Sector)) ) OR (((TI Health OR AB Health) W1 (TI Service?1 OR AB Service?1)) ) OR ((TI HCS OR AB HCS)) OR (((TI Medical OR AB Medical) W1 (TI Sector OR AB Sector)) ) OR ((TI Hospital?1 OR AB Hospital?1)) OR ((TI Clinic* OR AB Clinic*)) OR (((TI Nursing OR AB Nursing) W1 (TI home?1 OR AB home?1)) ) OR ((TI rehabilitation* OR AB rehabilitation*)) OR (((TI primary OR AB primary) W1 (TI care OR AB care)) ) OR (((TI speciality OR AB speciality) W1 (TI care OR AB care)) ) OR (((TI mental OR AB mental) W1 (TI health* OR AB health*)) ) OR (((TI public OR AB public) W1 (TI health* OR AB health*)) ) OR (((TI long-term OR AB long-term) W1 (TI care OR AB care) W1 (TI facilit* OR AB facilit*)) ) OR ((TI physician?1 OR AB physician?1)) OR ((TI nurs* OR AB nurs*)) OR (((TI allied OR AB allied) W1 (TI health OR AB health) W1 (TI profession* OR AB profession*)) ) OR (((TI medical OR AB medical) W1 (TI practitioner* OR AB practitioner*)) ) OR ((TI care* OR AB care*)) OR (((TI medical OR AB medical) W1 (TI device?1 OR AB device?1)) ) OR (((TI health OR AB health) W1 (TI informatics OR AB informatics)) ) OR ((TI patient?1 OR AB patient?1)) OR (((TI person?1 OR AB person?1) W1 (TI in OR AB in) W1 (TI need OR AB need) W1 (TI of OR AB of) W1 (TI care OR AB care)) ) OR ((TI invalid?1 OR AB invalid?1)) OR ((TI convalescent?1 OR AB convalescent?1)) OR (((TI sick OR AB sick) W1 (TI person?1 OR AB person?1)) ) OR ((TI elder* OR AB elder*)) OR (((TI elderly OR AB elderly) W1 (TI person?1 OR AB person?1)) ) OR (((TI elderly OR AB elderly) W1 (TI care OR AB care)) ) OR (((TI nursing OR AB nursing) W1 (TI home?1 OR AB home?1)) ) OR (((TI nursing OR AB nursing) W1 (TI personnel OR AB personnel)) ) OR ((MH "Health Care Sector")) OR ("Health Care Sector") OR ((MH "Health Personnel")) OR ("Health Personnel") OR ((MH Patients)) OR (Patients) OR ((MH Aged)) OR (Aged) OR ((MH "Health Services for the Aged")) OR ("Health Services for the Aged") OR ((MH "Nursing Homes")) OR ("Nursing Homes") OR ((MH Caregivers)) OR (Caregivers)
